# The Larynx the Source of the Vowel Sounds

**Published:** 1874-01

**Authors:** Thomas Brian Gunning


AMERICAN JOURNAL

OF

DENTAL SCIENCE.

Vol. VII. THIRD SERIES--JANUARY, 1874. No. 9.

### ARTICLE I.

### *The Larynx the Source of the Vowel Sounds.*

BY THOMAS BRIAN GUNNING, NEW YORK.

What I have observed in dental practice, associated with much special attention to speech, as heard in different forms of the mouth, from infancy to old age, not merely in normal conditions, but also in congenital and acquired deficiencies of the palate, and other defects, having convinced me that the source of the vowel sounds is misunderstood by those considered as authority upon the subject, I propose to show:

First, how the vowels are said to be formed in the mouth;
Second, that they cannot be formed in the mouth;
Third, where and how they are formed.

The human larynx divided into upper and lower cavities by the inferior or true vocal chords, and protected by its movable cover, the epiglottis, is well known to be the organ of voice. It is also understood that voice is produced by the air being forced from the lungs through the trachea

Entered according to act of Congress in the year 1873, by William Jessup Gunning, in the office of the Librarian of Congress at Washington.

and vocal chords, and that the pitch of the tones depends upon the vibrations of the vocal chords, as modified by their length, width, tension, etc., while the volume of the tone is regulated by the parts below.

It is also known that each tone of the voice has not only its fundamental tone, but that it includes also what are known as overtones, these being tones whose rates of vibration are twice, three times, four times, five times, etc.—that of the fundamental, and that different admixtures of the fundamental, and one or more of its overtones form the vowel sounds of speech and song. After much investigation and experiment by men of science, it has been decided that this change of vocal tones into the vowel sounds is made in the cavity of the mouth, say all parts of the vocal tube above the epiglottis. It is supposed that, by appropriate changes in these parts, the resonance of the mouth re-enforces the tone peculiar to the required vowel. The following references to what has been done and said by prominent investigators will make the views now held as to the formation of the vowel sounds more clear. In the year 1799 the Royal Academy of St. Petersburg made the vowel qualities a prize question. Kratzenstein submitted a memoir and also showed how the vowels could be produced by artificial mechanism, and the prize was awarded to him. Von Kempelen, of Vienna, made similar and more elaborate experiments. These scientists stated that the tones of the larynx were changed into vowel sounds by alterations in the size of the oral opening, and the oral canal, that is the aperture between the lips and between the tongue and palate. Mr. John Bishop in his work "On Articulate Sounds and on the Causes and Cure of Impediments of Speech," London 1851, page 17, says: "Vowels have been divided into three classes, having reference to the organs employed in their production, namely, guttural, palatal, and labial," and he endorses these views by explaining how the organs act, and brings in prominently certain vibrations of the lining menbranes of the parts which he considers to be specially active; in the gutturals, the membranes of the fauces, pharynx and soft palate; in the palatals, those of the dorsum of the tongue, and of the hard palate; in the labials, the vibrations in the lips and cheeks. This author also thinks that the change from the sound of *o*, as in bone, to *oo* as in boot, is entirely due to the closer and more projecting position of the lips, and he states distinctly that certain positions of the organs above the epiglottis are indispensable to articulate speech. He thinks that the vowel quality pulsations may be produced by either the air in the mouth, pharynx, and nostrils, or by the *membranes* of these parts; the first, being perhaps, the opinion of Dr. Thomas Young, the last, probably, more according to M. Savart. Mr. Bishop does not state clearly that he inclines to one view more than the other, but evidently thinks the vowel qualities originate in part from both sources.

Professor Tyndall, explaining the nature of the vowel sounds in his fifth Lecture on Sound, at the Royal Institution of Great Britain, published in New York, 1867, says: "We can distinguish one vowel sound from another while assigning to both the same pitch and intensity." * * * "Now, in the vocal organ of man, you have your reed in the vocal chords, and associated with this reed you have the resonant cavity of the mouth, which can so alter its shape as to resound, at will, either to the fundamental tone of the vocal chords or to any of their overtones. Through the agency of the mouth, we can mix together the fundamental tone and the overtones of the voice in different proportions, and the different vowel sounds are due to different admixtures of this kind." Professor Tyndall then selects a tuning fork of a suitable pitch for the required sound, and adjusts his mouth until it resounds to the fork, and then urges air through the larynx, when the particular vowel sound is heard and no other. In this way he decides that it requires the first, second, third, fourth, fifth and seventh tones to form all the different vowel sounds, upon the same fundamental tone of the vocal chords, and the precise tone or tones reenforced by the mouth for the particular sounds are for U (*oo* in hoop,) 1st or fundamental; O, 2d; A, 3d strong, 2d moderate, 4th and 5th weak; E, 1st weak, 2d comparatively strong, 3d feeble, 4th intense; Ah! 3d feeble, the higher tones especially, 5th and 7th strong. Upon this theory the mouth re-enforces tones ranging in pitch through three octaves less one tone to form the vowel sounds of speech on the same fundamental tone of the vocal chords, and this range of the mouth's re-enforcement must be increased in proportion to the range in the pitch of the tones of the vocal chords.

Madame Emma Seiler's views upon the formation of the vowel qualities are of great importance, from the fact that she was associated with Prof. Helmholtz in his experiments to ascertain the nature of these sounds. In the translation of her work, "The Voice in Singing," Philadelphia, 1870, she explains the nature of the vowel qualities, as explained by Prof. Helmholtz, and as just now given in the quotations taken from Prof. Tyndall's lecture. Madame Seiler, however, on page 96, says: "But certain vowels, in certain parts of the scale, can be sung far more easily and sweetly than others. The investigation of this fact has taught us that a tone gains in richness when the tone corresponding to the vowel belongs to the overtones of the fundamental tone." This seems to indicate that Madame Seiler considers that the vowel quality is given to the fundamental tones in a fixed range of pitch, and that it does not rise and fall with the pitch of the tones from the vocal chords, as Prof. Tyndall seems to intimate. Mme. Seiler's work shows uncommon acuteness and familiarity with the subject. She says: "The female voice has only a few tones more than an octave upon which every one of the vowels can be distinctly sung;" and on page 114, that "The length of the cavity of the mouth is the greatest in sounding *oo*, the least in *e*, intermediate in *a*." 'In the pure, clear *a*, as in *may*, the cavity is the narrowest.'

It is difficult to discover any practical difference between

Prof. Tyndall's and Mme. Seiler's views, and those of Mr. Bishop, upon the action of the organs in forming the vowel sounds of *speech.* They, however, seem to think, that the vowel qualities originate in the general resonance of the mouth, rather than in the vibrations in the membranes of any particular part of it. Many have written upon the vowel sounds of speech, but it is difficult to find in the writings of any one author, all that is necessary to give a comprehensive view of what is now believed in regard to them. This arises not so much from any material difference in the views held, as in the special subjects, upon which the writers were engaged. I have, therefore, selected from those whose writings collectively seem to embrace what will give an easy and sufficiently full idea of what is held respecting them. Mr. Bishop writes upon speech and its impediments ; Prof. Tyndall, more upon the strictly acoustic bearings of the subject ; Mme. Seiler, more upon the voice in singing.

Mr. Bishop describes minutely how the consonants are formed, and the positions taken by the organs of speech in doing this, have an important bearing upon the subject matter of this paper ; for the consonants are associated with vowels in the formation of words, and they have also their own special sounds readily distinguished, while the movements and positions necessary to their production are easily demonstrated.

I propose to show briefly some of the movements made in forming the consonants, and the probable effect of these upon the vowel sounds, if these last also originated in the mouth, but first giving a description of the parts used in speech above the epiglottis.

The lower jaw which protects the tongue, larynx and trachea, also gives attachment to muscles which control them in the performance cf their functions. Its co-operation in these functions is effected principally by the *temporal* and the *external pterygoid* muscles, the first pulling the upper part ot the ascending ramus backward, and the last drawing it forward. This shuts or opens the teeth and regulates the position of the lower jaw. Thus the *digastric* and other muscles attached to the body of the jaw, and to the hyoid bone, have a base of action, and without this, vocalization and articulation would be impossible. This will be more clearly understood by recollecting that with the teeth closed, the stylo maxillary ligament *slants* down to the angle of the jaw; therefore, while the *temporal* muscle relaxes to allow the *external pterygoid* muscle to draw the condyle forward under the *eminentia articularis*, the angle is, at the same time, carried back by the ligament, into the perpendicular of its attachment on the styloid process. This allows the jaw to go down bodily, as well as open in front, by which the back teeth of the upper and lower jaws are widely separated, and the size of the oral cavity much enlarged. The nose, with the nostrils through which the air passes to and from the pharynx at the back of the mouth cavity, and the .upper and lower lips, with the separation between them, which gives passage to and from the front of the mouth, are well known. The nose can vary the size and position of its anterior openings to a limited extent, but as a whole moves only with the head. The upper lip moves less on its attachment than the lower, this last moving freely with the lower jaw to the full extent to which this bone can carry it. These movements are much less in some persons than in others. The cheeks have also a varied amount of mobility, but yield readily to all the movements of the lower jaw. Each cheek is well controlled by a thin broad muscle, the *buccinator*, which arises from the alveolar process on each jaw, continues forward into both upper and lower lips, and backward on to the pterygo-maxillary lig ament, from whence on each side, the *superior constrictor*, a similar muscle, curves back to the centre of the pharynx. These four muscles lie just under the mucous membrane, which covers the inside of the cheeks, and all the parts within the mouth, except the teeth, continues up into the nose, and through the larynx into the lungs. The teeth which stand around within the lips and cheeks, are in the complete temporary set, twenty in number, and should be all in the mouth at three years of age. By six, the jaws have enlarged behind the last infant teeth, for the first permanent molars; by twelve, for the second; and by eighteen, the third permanent molars are generally present. Then the permanent teeth, thirty-two in number, should all be in the mouth, the cavity of which then averages four or five times larger than at the age of three. Its capacity ranges in the adult from two to four fluid ounces, males having the larger. Through early loss of teeth and other causes, the adult mouth has, in rare cases, less than two ounces capacity. I have never found one which could take in over four ounces of water without swallowing some. The separation between the lips varies much in size compared with the cavity within, and the hard palate, which forms the roof of the mouth, is singularly unlike in size and shape in different persons. The soft palate which extends backward from the hard palate, is complicated in its movements and requires close attention. It can be seen curving downward and showing in its centre the uvula which hangs below its back border. The uvula is the centre of two distinct arches, formed by two pairs of muscles, which are separated below by the tonsils. The posterior arch is smaller than the anterior, and therefore, also well separated from it above. It is formed by the *palato pharyngei* muscles, which by their anterior fibres go down and are inserted into the thyroid cartilage, while the others pass around the sides of the pharynx and some cross each other behind. The anterior arch is formed by the *palato-glossi* muscles, which are inserted into the tongue. The uvula is itself the insertion of a pair of small muscles, which pass back from the centre of the hard palate; while the *levator palati* muscle comes forward and downward over the edge of the superior constrictor on each side, and spreads out in the upper surface of the soft palate. This pair are the antagonists of those which pass to the uvula from the hard palate. The *tensor palati* of each side controls the tension of the soft palate. The form of the hard palate is such, that the tongue can close up against the edge around the inside of the upper teeth, while the back part of the tongue is so shaped, that it fits the uvula and soft palate exactly, and this closure can be made readily at the same time that the upper and back part of the soft palate also closes the posterior nares.

The tongue at rest lies with its point within the lower front teeth, rising gradually to its centre, where it curves suddenly down to its connection with the epiglottis, which fits up against it. Just below this the hyoid bone gives support to the tongue, and passes back around on each side, giving attachment by its upper and inner border, to a broad fibro-elastic membrane, which is also attached below around the upper border of the thyroid cartilage. From the back end of the hyoid bone on each side, a round elastic ligament also goes to the superior cornu of the cartilage. These attachments allow considerable movement between the hyoid bone and the thyroid cartilage. The tongue, above and in front of these, has singular mobility. Its point can be put out through the lips, up behind the teeth, against the gum, or drawn back in the mouth; it can also be turned down in front, below the back of the inferior incisors, while the centre goes up close into the roof of the mouth, and the part near the epiglottis is drawn so far forward, that the end of the finger may be placed between them, and this with the epiglottis up, and also pulled forward with the tongue. The tongue also, while fixed in the roof, can be drawn forward at its base so much that the epiglottis going forward with this part, is entirely protected by the tongue above it, as in swallowing. Other positions of the tongue are so easily seen, as not to require explanation here.

It is not surprising, with this wonderful flexibility of the tongue, and the complexity of the parts associated with it in speech, that even such a competent surgeon and so precise an observer as Mr. Bishop, should consider that the vowel sounds were formed in the mouth, and it requires close attention to arrive at a conviction that they are not.

It must be borne in mind that all words, whether in speech or song, without *m* or *n* in combination, are uttered with the soft palate drawn up so as to shut off the nose. This is easily proved by holding the nose shut with thumb and finger, when after sounding any letter of the alphabet other than *m* or *n*, the opening of the soft palate will be heard and felt. It comes down sooner in some sounds than in others. In singing low tones, as seen in the mouth, it appears to be open, but this is so only in its lower border, and in sounding low tones, with the nose closed, it takes much longer to open than in high tones, in which the velum is drawn up tight, and the tension of the parts then opens it quickly. The velum is drawn up back by the *levators*, which enter the palate above. The small muscles between the spine of the hard palate and uvula, draw the velum down in a curve to fit the tongue, and if the finger is laid upon the tongue so as to press it down where the uvula strikes it in swallowing, or in sounding the guttural consonant *k*, the uvula, if short, will come forward and its back lay upon the finger; while the tongue and velum are in contact to sound *k* or *g* hard, and kept so, the nose can be opened instead of sounding either letter and the sudden current of air will prove that it was firmly closed behind, and this closure is complete even with the larynx prepared to sound the lowest tones of the voice. This isolation of the nose, except in sounds of *m* and *n*, makes it unnecessary to give any special attention to the resonance of its cavity.

Mr. Bishop defines the guttural consonants to be *c* and *g* hard, *k*, *q*, *ch*, *gh*, and says: "The vowel sounds pronounced in *ball*, *bar*, *bat*, *but*, are guttural."

He says *t*, *d*, are lingua-palatal consonants, and the vowels in *bate*, *bet*, *beat*, *bit*, palatal.

He says *b*, *p*, are labial consonants, and the vowel sounds in *bone* and *boot*, labial.

These different sounds are formed upon a tone originated by the vibration of the vocal chords, this tone not being at all altered in pitch, but only so that the vowel quality is given to it, and so far as the consonants as used in speech, are more than the mere beginning or ending of a syllable or word, they consist of some vowel sound, and this, although slight, is readily distinguished, for *t* or *k*, in combination with the same vowel are not confounded, although both are formed out of sight.

Now, if the consonants and vowels, classed as guttural, were sounded only with each other, it would be easier to believe in Mr. Bishop's theory; but the guttural consonants are used quite as well with the palatal and labial vowels, and the labial and other consonants are also used with all the vowels. In fact, while any consonant is used with any vowel, consonants of the different classes are used with the same vowel, or the same vowel sound, in syllables and words having one continuous vowel sound. For instance, in sounding *cop*, there is no alteration of the vowel sound, but on Mr. Bishop's theory, *cop* could not be sounded at all, for in passing to form the labial *p*, it would end as *coop*; the word *cob*, would be *co-oob* as the lower lip closed to form b.

I have thus far confined myself to *o* and *oo*, as Mr. B. says the transition from one to the other is entirely due to the change in the state of the lips. Now, if it be said, that state of the lips may be more than position, that it may also include vibration—take the word *cove.* Here the lip in closing up to form the *v*, also vibrates, but although it goes up past the position for *oo* and then rests in it, the word gives no sound of *oo* whatever. It is clear therefore, that something more than position, or *state* of the lips, is needed to make the difference between *o* and *oo*.

These examples could be multiplied, but without advantage. We know how and where the consonants are formed, and in the transitions from one to the other, it is impossible that a pure vowel sound could continue unchanged, were they formed in close proximity, indeed in the mouth at all. Mr. Bishop himself says: "Diphthongs are not merely sounds resulting from the combination of two simple vowels following each other in rapid succession, but are also in part the effect of the movements of the articulating organs on the vowel sounds, during the transition of these organs from the position of the first vowel to that of the second." Now if the vowel sounds originated in the mouth, during transition of the organs to form different *consonants*, the continued vowel sound between them would be altered. With these movements it is impossible that vibration of the parts designated can add the vowel quality to the fundamental tone of the voice, and also hold the vowel sound steady and pure. Further, the vowel sound may be formed well, although the fingers are against the teeth in place of the lips, and also in cases of congenital cleft palate, where not only the soft but the hard palate, and even the upper lip is deficient. Of course in these cases the sound is more or less nasal. But this is remedied by a plate of hard rubber, which covers the whole of the roof of the mouth, passes up above the remnants of the soft palate, fills up all across, and ends in the pharynx opposite the tubercle of the atlas. By this, vibrations of the hard and soft palate and nearly the whole of the pharynx are cut off, but the vowel sounds are perfect, and the consonants so far, that the imperfect condition of the palate is not suspected; and there can be no vibration whatever in the hard rubber. The vowels are perfect when all the gum of both jaws, and the roof of the mouth are covered with plates holding artificial teeth, the plates being of hard rubber, metal, or porcelain, and vibrations in, or beneath them impossible. Nor is the vibration, nor in fact the presence of the tongue indispensable; for the records are indisputable which show that the loss of the tongue is not followed by loss of speech, and that the *vowel* sounds are articulated perfectly with the tongue cut off nearly against the epiglottis.

It is necessary to consider carefully, at this point, what this change of tone to vowel sound is in acoustic quality, or value. This is not definitely settled. Mr. Willis,* quoted

* Cambridge Philosophical Transactions, Vol. iii. 1829.

largely by Mr. Bishop, and referred to by Prof. Tyndall, makes the difference in pitch between the vowel quality in *ee*, as in *see*, and *o*, as in *no*, to be three octaves and a fifth ($g''''$ to $c'$) marking that in the lowest vowel sound *oo*, as in *boot*, indefinite. This would make a difference of, say, four octaves between the highest and lowest vowel quality. He used a reed which sounded the same fundamental, and the pipe which surmounted the reed and resounded to it, had always the same outlet, but was varied in length to make it yield the vowels, the length for *ee* being .38 of an inch, and for *o*, 4.7 inches,— over twelve times longer. This difference in the depth of the resonant cavity would be increased to sixteen times for *oo*, the lowest vowel quality, if this vowel sound were just four octaves below *ee*,—the intermediate vowels being obtained between these extremes.

Prof. Helmholtz gives a scale of four octaves less a tone and a half, in which the overtones of the vowels as pronounced in the north of Germany are particularly strong.

Dr. C. L. Merkel, of Leipzig, gives a range of three octaves, less two and a half tones, to the pitches of the vowels, according to his own habits of speech.

Prof. Tyndall says it requires three octaves less one tone to form the vowel sounds upon the same fundamental, and this range must be increased as far as the tones of the vocal chords range. Now the range of the voice in speaking and singing, being, say, two octaves, full two-thirds of this range of the fundamental tones, and their overtones should be so re-enforced by the mouth as to form perfect vowel sounds. To do this, every mouth must alter so as to augment tones ranging through four octaves and a third; and as the male voice is an octave lower than the female, the range of tones necessary to be re-enforced by the mouth in both sexes is over five octaves. For the purpose of this paper, however, it is not important that the scale of the vowel qualities should be precisely known.

In the mechanism used by Mr. Willis, to find the acoustic values of the vowel qualities, the pipe was necessarily greatly varied in length. This presents, at once, an insurmountable objection to the theory that the vowel qualities of speech originate in the cavity of the mouth, which has little capability of altering its length. In other respects, however, the acoustic possibilities of the mouth, are not so easily settled, or the matter would have been decided long ago.

In Willis' experiment we see that the resonant cavity of the pipe is longer, compared with its outlet, in proportion to the gravity of the tone, and shorter as the tone becomes acute. It may, therefore, be thought that the equivalent of this change in the resonant cavity, would be found by varying the size of the outlet, the cavity remaining the same. My own experiments show, however, that in a glass bottle having a mouth about one inch in diameter, partly filled with water, leaving a space of four and a quarter fluid ounces capacity, the cavity resounded to *c''*; with the mouth half closed, *b'* flat; while *g'*, only two tones and a half below *c''*, would not resound at any closure.

To show the effect of lessening the resonant cavity, while retaining the same opening, $3\frac{1}{2}$ ounces of space gave *e''*; $2\frac{1}{2}$, *g''*; $1\frac{5}{8}$, *c'''*. This $3\frac{1}{2}$ ounces of space is about the capacity of a large adult mouth, yet this space reduced more than one half, by pouring in water, resounded to a pitch only four tones, or two-thirds of an octave higher. This arose from the water not filling up half the length of air space, as the bottle was wider below than at its neck. In a straight tube to fill half the space, would halve the length, and the empty part would resound to an octave higher.

Four straight jars without necks, their capacities being respectively $1\frac{1}{2}$, $2\frac{3}{4}$, $4\frac{1}{2}$, and 9 fluid ounces, covered with the same sheet of gutta-percha, with an opening in the centre about the shape and size of a small mouth, the opening, that is, between the lips responded to *c'''*, *g''*, *e''*, *b'* flat. This is a difference of only one whole tone more than an octave, between the largest and smallest jars, and yet the large jar was three inches deep—just twice the depth of the small one, and six times its capacity. The three smallest jars differ in size far more than any cavity of the mouth, from any possible alteration made by the tongue in speech; yet their whole range of pitch was but four tones, and even this was caused by the difference in their depth of air cavity, which was far greater in any two of them than is possible to the cavity of any one mouth in speech.

Length, or depth, has more influence on pitch, than mere size of cavity, yet a proportionate size is also necessary; for a wide mouthed bottle filled with water to three inches from the top, and resounding to *c''*, on being laid down so far that the water came just to the edge of the mouth, by which the space was doubled in length, but still the same in size, resounded to only one tone lower, not the octave lower, as it would had the space been doubled in size, as well as in length, instead of tapering. Again a straight tube, with space four inches deep, and of 2¾ fluid ounces capacity, resounded to *e''*; and on laying it down as above, to *c''*, two tones lower. These resonant cavities were respectively three and four inches deep, while the mouths of adults, between the back of the pharynx, and the closing ridge of the lips, generally range from three and a quarter, to three and three-quarter inches, rarely being as short as three or as long as four.

By these tests of mere resonance made, like Prof. Tyndall's, at the outlet of the cavity it is seen that *altering the size of the outlet* affords but a very limited range in the pitch of the tone; further that a cavity of a certain size, *double in length, but without increased capacity*, varies only a little in its tone or key note, and finally that changing the size of the resonant cavity without proportionate *increase of length in one direction*, has also but little effect on pitch. The mouth has only the most limited power to lengthen its cavity, and this is confined to the lips, for the constrictor muscles which give form to the sides and back of the pharynx, are firmly held in the centre behind. These experiments show therefore that the human mouth cannot form the vowel qualities, by altering its outlet between the lips, its shape, or its capacity.

The remarks previously made as to the effect of cleft palate, and imperfect lips, loss of the tongue, and other acquired injuries, are equally pertinent here; for any one of them would prevent the resonance of the mouth from forming the vowel qualities; and as the vowels are comparatively perfect in such cases, it is clear that they originate elsewhere. In fact, if they did not, speech would be impossible, for the changes in the organs to form even the consonants, would prevent a continuous and pure vowel sound in any syllable into which they entered, if the resonance of the mouth had any considerable influence.

Further, the changes in position of the organs, as seen in singing, show plainly that they act in subordination to the general pitch of the voice as a whole, and not for the addition of the vowel qualities. In high tones the mouth opens wide; in low tones the lips are comparatively close; while the soft palate is high and tense in acute tones, but low and loose in grave tones; and these great changes, so plainly seen, are made to enable the mouth to transmit the tones of the larynx, which with all its special parts for forming the voice, and varying its pitch, gives less than two octaves range to the average of voices, as used in singing, or far less than the range of pitch necessary in the vowel qualities, to form the vowel sounds of any one person.

It is thus clearly seen that the mouth is supplied with the vowel sounds already formed upon the tones of the larynx. I purpose to show now, that the addition of the vowel qualities to the tones of the vocal chords is made in the upper cavity of the larynx. This cavity is bounded below by the true vocal chords, which pass back in the thyroid cartilage from their attachment on each side of the centre, corresponding to the vertical ridge so prominent and easily seen in the male larynx in front of the throat. The vocal chords are attached in the inside, a little above the small membrane felt as a notch in the front of the neck, at the inferior border of the thyroid cartilage. The false vocal chords are close above; and, higher still, at the inside of the extreme point known as the *pomum Adami*, the ligament of the epiglottis is attached. From this point, the upper edge of each side of the cartilage may be felt, curving upward and backward, and if the finger is laid on the membrane between, the body of the hyoid bone will be felt above, as a hard round projection in the soft parts within the lower jaw. The support given by this bone to the larynx by means of this membrane, which passes down around to the upper border of the thyroid cartilage, has been before spoken of. The epiglottis passes up from its ligamentous attachment to the thyroid cartilage, and is attached by an slastic ligament to the inside or posterior surface of the body of the hyoid bone, after which it emerges at the back of the tongue, as a broad elastic cover to the superior aperture of the larynx. The tongue, as before stated, commences at, and rests upon the top of the hyoid bone. This bone, however, is not the only connection between the tongue and epiglottis, as three folds of mucous membrane also link them together in the mouth. The point of the thyroid cartilage in front of the throat, known as the *pomum Adami*, is some distance below the hyoid bone, but in swallowing it may be felt to rise up close to the bone, and then go forward with it toward the inside of the chin. This is the highest position which the cartilage can reach, and, by following it with the finger, we feel that the upper cavity of the larynx is subjected to great changes in shape and capacity. During the act of swallowing it may be noticed also, that the tongue is fixed in the roof of the mouth, and the hyoid bone, therefore, in going forward, draws the larynx, epiglottis and base of the tongue under the part above. By this, the epiglottis, which is usually upright, is carried down backward over the superior aperture of the larynx, which is thus closed, and well protected while food passes down behind. With the finger upon the thyroid cartilage, it may be felt that its sides widen rapidly from the *pomum Adami* in front, and, as the vertical ridge in front recedes down to the lower border, and the upper borders of the thyroid cartilage, with the membrane between, (which, with the attachment of the epiglottis extend up to the hyoid bone,) also recede, it follows that the inner surfaces of the front of the upper cavity of the larynx, converge to the inside of the point of the *pomum Adami.* The upper surface, however, along the course of the piglottis, and the aperture, as well, which the latter modifies, vary considerably in deglutition, vocalization and ticulation. In movements of the tongue outside of these functions, the hyoid bone, although supporting the tongue, changes but little in relative position to the thyroid cartilage, and in mere movements of the jaw to open the mouth, the hyoid bone, and the thyroid cartilage are virtually motionless. Consideration of these statements of the movements in deglutition will assist in discriminating between them, and those of vocalization and articulation yet to be considered. If the arm is laid upon the breast, with the forefinger resting on the membrane between the superior borders of the thyroid cartilage, and its end against the hyoid bone, while the second finger and thumb lay hold of the cartilage below the *pomum Adami*, any movement in these parts will be distinctly felt. The hand may be steadied equally by resting the knuckle of the forefinger against the chin above. Allowance must be made in the latter position for movements of the jaw in opening the mouth, and in the former for movements of the breast in breathing.

TO BE CONTINUED.

